# Supplementary material for: Chromothripsis during telomere crisis is independent of NHEJ, and consistent with a replicative origin
Source: Genome Res. 2019 May;29(5):737–49. doi: 10.1101/gr.240705.118 (PMC6499312; doi:10.1101/gr.240705.118)
Supplement: Supplemental Material [file supp_gr.240705.118_Supplemental_file_1.zip › contigs/annotated_contigs/DB57/contig.2.DB57_length_686_mean_cov_10.9183673469.docx]

**DB57_length_686_mean_cov_10.9183673469**

```
AAATAACACTAGAGCACATAATTAACAACCTGAACAAACTAAATTCAGTTTTACATCAAGACATATGGAAAAATAAATTTAAATTCAAG
 >chr5:84778032-84778360 + E=1e-182 p=2e-02
GAAACTATTAAAAAGTTAAACAAAACATACTAACCAGAAGTACAGGTTTACTTAAAAGTTTTTGGACAAAATGTCTGCACGAATATGTG

CCTGTATGTGTTAAATAATCCCAATGTAGCGTTTAATAATATTAAAAAACATTTTACTCATCTAATTGGCACAGTTTAAATATTGATAG

TATCCAGCATTAGGATGAAAAGAAAGAAGTGGGAGTTCTCATGTACTTCTGATTGGATT|AA|AATAGCTGCTCCATCTGGTAAGAAGG
                                                            >chr11:46093607-46093967 -
GGGAGAAGCAACAAAGAGCAAGCCCTTTATTTTTTAAGGATGTTTACTAGAAGTTGCATGCACCACTTCCAGTTAGATCCCATGGGCCT
E=7e-205
GAACTTAGTCATGTGGCCCCTGCTAGGAGCAAGGAAGTCTGGAAAATGTAGGTAGTTTATTGCTGGTAGTCATGGCCAGATAAAATTCA

AGGACTATTACTGAGTAAGAATTGGGAAATAAATACTGGGAATAATTAGCATTTTCTATTACAGTAGCATATCTGTATCTGTAGGATTA

CTATTTTTTAAATATCTGTCTTCCTTATTAGATTGTTAGTTTCAGGAAGTCAGGGAACGTGTTTG
```
